# Hot and Hypoxic Environments Inhibit Simulated Soccer Performance and Exacerbate Performance Decrements When Combined

**DOI:** 10.3389/fphys.2015.00421

**Published:** 2016-01-12

**Authors:** Jeffrey W. F. Aldous, Bryna C. R. Chrismas, Ibrahim Akubat, Ben Dascombe, Grant Abt, Lee Taylor

**Affiliations:** ^1^Department of Sport Science and Physical Activity, Institute of Sport and Physical Activity Research, University of BedfordshireBedford, UK; ^2^Sport Science Program, College of Arts and Sciences, Qatar UniversityDoha, Qatar; ^3^Department of Physical Education and Sports Studies, Newman UniversityBirmingham, UK; ^4^Department of Rehabilitation, Nutrition and Sport, School of Allied Health, La Trobe UniversityMelbourne, VIC, Australia; ^5^Department of Sport, Health and Exercise Science, The University of HullHull, UK; ^6^ASPETAR, Qatar Orthopedic and Sports Medicine Hospital, Athlete Health and Performance Research Centre, Aspire ZoneDoha, Qatar

**Keywords:** decrements, football, hot, hypoxia, physical, physiological

## Abstract

The effects of heat and/or hypoxia have been well-documented in match-play data. However, large match-to-match variation for key physical performance measures makes environmental inferences difficult to ascertain from soccer match-play. Therefore, the present study aims to investigate the hot (HOT), hypoxic (HYP), and hot-hypoxic (HH) mediated-decrements during a non-motorized treadmill based soccer-specific simulation. Twelve male University soccer players completed three familiarization sessions and four randomized crossover experimental trials of the intermittent Soccer Performance Test (iSPT) in normoxic-temperate (CON: 18°C 50% rH), HOT (30°C; 50% rH), HYP (1000 m; 18°C 50% rH), and HH (1000 m; 30°C; 50% rH). Physical performance and its performance decrements, body temperatures (rectal, skin, and estimated muscle temperature), heart rate (HR), arterial blood oxygen saturation (S_a_O_2_), perceived exertion, thermal sensation (TS), body mass changes, blood lactate, and plasma volume were all measured. Performance decrements were similar in HOT and HYP [Total Distance (−4%), High-speed distance (~−8%), and variable run distance (~−12%) covered] and exacerbated in HH [total distance (−9%), high-speed distance (−15%), and variable run distance (−15%)] compared to CON. Peak sprint speed, was 4% greater in HOT compared with CON and HYP and 7% greater in HH. Sprint distance covered was unchanged (*p* > 0.05) in HOT and HYP and only decreased in HH (−8%) compared with CON. Body mass (−2%), temperatures (+2–5%), and TS (+18%) were altered in HOT. Furthermore, S_a_O_2_ (−8%) and HR (+3%) were changed in HYP. Similar changes in body mass and temperatures, HR, TS, and S_a_O_2_ were evident in HH to HOT and HYP, however, blood lactate (*p* < 0.001) and plasma volume (*p* < 0.001) were only significantly altered in HH. Perceived exertion was elevated (*p* < 0.05) by 7% in all conditions compared with CON. Regression analysis identified that absolute TS and absolute rise in skin and estimated muscle temperature (*r* = 0.82, *r* = 0.84 *r* = 0.82, respectively; *p* < 0.05) predicted the hot-mediated-decrements in HOT. The hot, hypoxic, and hot-hypoxic environments impaired physical performance during iSPT. Future interventions should address the increases in TS and body temperatures, to attenuate these decrements on soccer performance.

## Introduction

Environmental stress in elite soccer is an important consideration for both practitioners and policy makers (Taylor and Rollo, 2014). Indeed, eight of the last 19 Fédération Internationale de Football Association (FIFA) World Cups were hosted by countries located at either low (500–2000 m) or moderate (2001–3000m) altitudes (e.g., 2010 FIFA World Cup, South Africa, 1200–1700 m; Bartsch et al., 2008; Billaut and Aughey, 2013). Specific to the Union of European Football Associations (UEFA) region, fixtures are often played above sea level (e.g., Molde, Norway, 1000 m) and/or in hot environments (e.g., Madrid, Spain, 30°C—Taylor and Rollo, 2014). In relation to heat-stress, temperatures often exceeded 30°C (Maximum: 35°C) in the 2014 FIFA World Cup hosted by Brazil (Nassis et al., 2015). Furthermore, combinations of both high temperature and altitude (hypoxia) can be experienced during elite soccer match-play (e.g., Saint-Etienne, France, 30°C; 1000 m).

Soccer match-play data indicates a decline in physical performance in both heat (Ekblom, 1986; Mohr et al., 2010, 2012; Özgünen et al., 2010) and hypoxia (Aughey et al., 2013; Nassis, 2013; Garvican et al., 2014; Buchheit et al., 2015) due to a complex interplay between peripheral, central and perceptual mechanisms (Nybo and Secher, 2004; Billaut and Aughey, 2013; Goodall et al., 2014; Nybo et al., 2014). However, the combined permutations of heat and hypoxia during match-play have not been investigated, although logically their combination would likely exacerbate physical performance decrements. At 43°C (Mohr et al., 2012), total distance (−7%), and high-speed distance (−26%) covered are reduced, with these changes being attributed to a multitude of proposed mechanisms including increasing body temperatures (Nybo et al., 2014). Furthermore, alterations in tactical behavior (e.g., reduced pressing of the ball) has meant that sprint distance covered is unchanged and peak sprint speed is enhanced during heat-situated soccer match-play (Özgünen et al., 2010; Mohr et al., 2012; Taylor and Rollo, 2014; Flouris and Schlader, 2015). Soccer match-play at low altitudes [1200—(Nassis, 2013); 1600 m—(Garvican et al., 2014) above sea level] leads to a decline in total distance (3.1%) and high-speed distance (15%) covered as recovery from high-speed intermittent activity is prolonged, due to the onset of exercise-induced-arterial-hypoxemia caused by a reduction in partial pressure of oxygen within the atmosphere (Billaut and Aughey, 2013). However, sprint performance is enhanced in hypoxia due to improved aerodynamics and flight time of an athlete through the air (Levine et al., 2008), highlighting that different components of soccer performance (e.g., sprint performance) are likely to respond differently within heat and/or hypoxia (Mohr et al., 2012).

Soccer match-play data, including key physical performance measures (e.g., high-speed distance covered), shows high match-to-match variation due to a plethora of match factors, such as tactics, score, opposition, etc. (Gregson et al., 2010). This variability in key physical performance measures may be exacerbated in both heat (Mohr et al., 2010, 2012; Özgünen et al., 2010; Nassis et al., 2015) and hypoxia (Aughey et al., 2013; Nassis, 2013; Garvican et al., 2014; Buchheit et al., 2015) resulting in an altered “pacing strategy” and exercise intensity (Taylor and Rollo, 2014). Recently, Gregson et al. (2010) suggested that to obtain meaningful inferences from a soccer match-play research design, a minimum sample size of 80 players would be required. Consequently, it appears that the majority of match-play based studies examining environmental influences on soccer performance are underpowered (<25 participants; Özgünen et al., 2010; Mohr et al., 2012; Aughey et al., 2013; Garvican et al., 2014; Buchheit et al., 2015), compared to the sample size (*n* = 80) proposed by Gregson et al. (2010). Only two studies have utilized an appropriate sample size (>*n* = 80) to assess the performance decrements associated with soccer match-play in hypoxia (Nassis, 2013) and heat (Nassis et al., 2015) during the 2010 and 2014 FIFA World Cup's, respectively. In particular, Nassis et al. (2015) revealed that in hot environments, players preserved key physical performance measures (e.g., peak sprint speed) that are associated with the match outcome (Faude et al., 2012), by reducing the number of sprints and high-speed efforts performed during a match. However, irrespective of the environment, players are likely to modulate their physical performance to avoid an earlier onset of fatigue during a tournament (Dellal et al., 2013), making environmental-mediated-inferences difficult to ascertain from the international tournaments data (Nassis, 2013; Nassis et al., 2015).

Recent reviews (Taylor and Rollo, 2014; Roelands et al., 2015) have recommended a solution to this “sample size issue” is to utilize an individualized, valid and reliable soccer-specific simulation to quantify environmentally-mediated performance decrements with greater experimental control. Aldous et al. (2014) demonstrated that the intermittent Soccer Performance Test (iSPT) is a valid, reliable and individualized (i.e., individualized speed thresholds) laboratory and non-motorized treadmill (NMT) based soccer-specific simulation; which can ascertain changes in soccer performance more robustly compared to match-play data with limited sample sizes. By utilizing iSPT, changes in soccer performance between the identified conditions (e.g., hot and/or hypoxic) can be determined in a controlled environment, minimizing match factors (Gregson et al., 2010) and the within game (Mohr et al., 2005, 2010) and tournament (Dellal et al., 2013) enforced pacing strategies (Nybo et al., 2014; Périard and Racinais, 2015; Roelands et al., 2015), unlike previous environmentally-situated match-play derived data (Mohr et al., 2012; Garvican et al., 2014).

Therefore, the aim of this study was to utilize the iSPT to reliably quantify soccer performance in hot (HOT), hypoxic (HYP), and hot-hypoxic (HH) environments (Aldous et al., 2014). The first experimental hypothesis was that physiological strain would be increased in HOT, HYP, and HH compared with CON, causing a significant reduction in physical performance in HOT, HYP, and HH. The second experimental hypothesis expected the hot and hypoxic environments to enhance sprint performance in HOT and HYP. Finally, the third experimental hypothesis was that in HH, physiological strain would be exacerbated compared with HOT and HYP causing a larger decline in physical performance.

## Methods

### Participants and experimental controls

Twelve male, University level soccer players [median (min-max) age = 23 (18–33) y; mass = 77 (67–93) kg; height = 1.81 (1.68–1.95) m; mean ± SD $\overset{{}^{\circ}}{\text{V}}{\text{O}}_{2\text{max}}$ = 57 ± 2 mL^.^kg^−1.^min^−1^] volunteered for this study. An a *priori* power calculation (G*Power 3) was used to determine the number of participants required for this experiment (*n* = 12) with an alpha level of 0.05 and a statistical power of 99%, using data [(high-speed distance covered)—minimum worthwhile effect = 5 m; SD = 50] from Aldous et al. (2014). All participants were members of the University of Bedfordshire Soccer team who trained at least two times per week and played at least one full 90 min match per week. The study was approved by the University of Bedfordshire Ethics Committee, and conformed to the declaration of Helsinki. All participants were fully informed of the risks associated with this study before they gave full written consent to take part in testing. Participants standardized their food and water consumption (Sawka et al., 2007) and abstained from alcohol, cigarettes, caffeine, and strenuous exercise at least 48 h prior to testing and maintained their normal diet prior to and during the testing sessions (in line with Taylor et al., 2014). Participants refrained from supplementation of ergogenic aids throughout the study and had not been exposed to >30°C and/or >1000 m above sea level three months prior to this study (Taylor et al., 2010). Adherence was assessed by questionnaire, with no violations seen for these control parameters.

Participants were instructed to drink 2–3 L of water 24 h prior to all laboratory visits (Sawka et al., 2007; Taylor et al., 2012) as prior to each experimental trial hydration status was assessed via urine osmolality (Atago-Vitech-Scientific, Pocket-PAL-OSMO, HaB-Direct, Southam). Euhydration was deemed when urine osmolality was below 600 mOsm/l (Hillman et al., 2013). Testing times were held constant for individuals due to the effects of circadian variation upon rectal temperature (*T*_re_; Racinais et al., 2012) and physical performance (Drust et al., 2005).

### Study design

All familiarization (FAM), peak speed assessments (PSA), and iSPTs were completed on the same NMT (Force 3.0, Woodway, Cranlea, Birmingham).

#### Visit 1–3 (FAM_1-3_ and PSA)

The three FAM sessions and one PSA session were completed as per Aldous et al. (2014). FAM_1-3_ robustly familiarized [as demonstrated by Aldous et al. (2014)] participants to iSPT and the running mechanics of NMT locomotion, which compared to “free” running and motorized treadmill running has notable differences (Lakomy, 1987). Familiarized participants (i.e., post FAM_1-3_) subsequently (1 h post-FAM) completed a PSA, which identified each participant's familiarized peak sprint speed. The PSA derived of four 6 s maximal sprints over a 4 min period with equal rest (1 min) between sprints to allow adequate recovery time. For each participant, the peak sprint speed was defined as the fastest speed recorded during the PSA. The peak sprint speed was then utilized to individualize all speed thresholds during iSPT to each participant (Aldous et al., 2014; Coull et al., 2015). So for example, a participant with a peak sprint speed of 24 km·h^−1^, would have the following speeds to achieve for each movement category across iSPT; stand (0 km·h^−1^), walk (5 km·h^−1^), jog (8 km·h^−1^), run (12 km·h^−1^), fast run (14 km·h^−1^), and sprint (24 km·h^−1^). The percentage of peak sprint, ascertained from the PSA, and how this determines the required speed for each movement category across iSPT is detailed in Table 1. These speed thresholds determined the speed (target speed/threshold) participants had to obtain for each movement type (stand, walk, jog, run, fast run, and sprint). The frequency and distribution of these movement types (Table 1) were based upon the findings of previous match-play data, and were shown by Aldous et al. (2014) to be valid and reliable. No other physical performance measures were calculated during the FAM and PSA sessions. *Visits 4-7*: A randomized-controlled design was then used with each participant completing four experimental trials of iSPT: CON [0 m; 18°C, 50% Relative Humidity (rH)], HOT (30°C; 50% rH), HYP (1000 m; 18°C 50% rH), and HH (1000 m; 30°C 50% rH). All experimental trials were separated by 7 d and completed within a controlled laboratory environment (Flower-House, Farm-House, Two-Wests and Elliot, Chesterfield) where hot and hypoxic exposures were administered using a portable heater (Bio-Green, Arkansas-3000, Hampshire) and an adjustable hypoxicator (Everest-Summit-II, The Altitude Centre, UK), respectively. The adjustable hypoxicator mask was worn in all four experimental trials. Environmental temperature, rH and simulated altitude were measured continuously during all experimental trials (Table 2). Prior to completing iSPT, all participants completed a 10 min warm up on the NMT at a speed of 8 km·h^−1^ and including 2 brief sprints (<4 s), as per Oliver et al. (2007). The 10 min warm up took place in the subsequent environment each experimental condition was performed in.

**Table 1:** **The percentage of intensity, frequency, and total time spent at each movement category during iSPT (obtained from Aldous et al., 2014)**.

**Movement Category**	**% of PSS**	**Frequency**	**Total Time (s)**	**Total Time (%)**
Stand	0	240	1920	17.8
Walk	20	456	3936	36.4
Jog	35	300	2592	24.0
Run	50	192	1248	11.6
Fast run	60	72	384	3.6
Variable run	Unset	48	288	2.7
Sprint	100	72	432	4.0
Total	—	690	5400	100

*PSS, Peak Sprint Speed; s, seconds; %, Percentage*.

**Table 2:** **The environmental conditions simulated during this study**.

**Environmental Condition**	**Temperature (°C)**	**rH (%)**	**Altitude (m)**
CON	18.1 ± 0.6	50.8 ± 0.6	0 0 ± 0.0
HOT	30.3 ± 0.5	50.3 ± 0.3	0 0 ± 0.0
HYP	18.2 ± 0.9	50.3 ± 0.6	1.001 ± 10.9
HH	30.5 ± 0.8	50.5 ± 3.6	1.003 ±10.5

*CON, Normoxic-Temperate; HH, Hot-Hypoxic; HOT, Hot; HYP, Hypoxic*.

#### Intermittent soccer performance test

**Figure 1:**
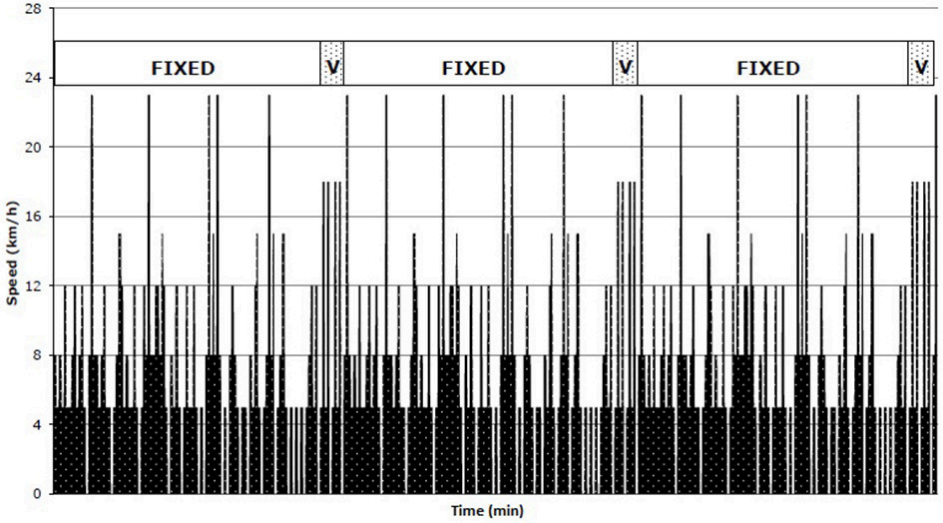
**The 45-min activity profile of iSPT for a participant with a peak speed of 23 km·h^**−1**^ (obtained from Aldous et al., 2014)**.

The iSPT consisted of two 45 min halves comprised of three identical 15 min intermittent exercise blocks (Figure 1; Aldous et al., 2014), utilizing the movement categories detailed previously (stand, walk, jog, run, fast run, variable run, and sprint). The frequency and durations of these movement categories (and how their respective target speeds/thresholds are calculated) across the iSPT are provided in Table 1 with an example provided within the previous section. Throughout each 15 min block for all target speeds apart from the variable run, participants interacted with a computer program (Innervation, Pacer Performance System Software, Innervation, Pacer Performance System Software, Lismore, Australia) by following a red line on the screen (which displayed their target speed) and their current (actual) speed (green line). If a discrepancy between target and current speed (i.e., the lines did not closely overlap) was evident participants had to run more quickly, or slowly, accordingly, to realign the lines. Participants were instructed to match their current speed with the target speed as closely as possible throughout iSPT for all target speeds related to each movement type (stand, walk, jog, run, fast run, and sprint) apart from the variable run. Audio cues specific to each movement category (e.g., jog) were also presented. Before each change in speed, three audible tones were played, which were followed by an audible command to inform the subject of the upcoming activity (e.g., “beep,” “beep,” “beep,” “run”). Four self-selected high-speed runs (variable run: 13–14th min of each 15-min block; Figure 1) were included, where the participant was instructed to cover as much distance as possible without sprinting.

### Physical performance measurements

Data for total distance covered was comprised from all movement categories and was calculated between both halves and conditions. High-speed, variable run, and sprint distance covered was computed for each half and entire condition as well as the total amount performed in each 15 min block (Aldous et al., 2014). Peak sprint speed was only obtained as the fastest speed seen for each 15 min block. Performance decrements for all physical performance measurements were calculated in distance covered (m) and percentage (%) between conditions halves and 15 min blocks.

### Physiological measurements

Prior to the FAM, height (cm) was measured using a Holtain Stationmaster (Stadiometer, Harpenden, HAR 98.602, Holtain). Body Mass (kg) was also measured pre- and post-iSPT using digital scales (Tanita, BWB0800, Allied Weighing) to account for the fluid loss for each player, with the 500 mL of water players consumed during the half-time break accounted for. Heart rate (HR) was recorded beat-by-beat and averaged every 1 min using a telemetric heart rate monitor (Polar, FS1, Polar Electro, Oy). Fingertip blood samples were taken to assess blood lactate (Bla; YSI, 2500 stat plus, YSI) during walking or standing phases of the iSPT at 12, 27, and 45 min of each half (Aldous et al., 2014). All Haematocrit (Hct) samples was collected into heparinised capillary tubes (Hawksley & Sons Ltd, UK) and then centrifuged at 5000 RPM for 3 min (Hawksely, Micro Haematocrit centrifuge, Hawksley & Sons Ltd, UK). The Hct levels were read from the Haematocrit reader (Hawksley, UK). Hemoglobin (Hb) concentration was then collected via micro-cuvettes (Hemocue, Hb 201, Hemocue Ltd, Sweden) and then measured using a B-Hemoglobin photometer (Hemocue, Hb 201^+^, Hemocue Ltd, Sweden).

Changes in blood plasma volume (%ΔPV) both within/between tests were then estimated from Hb and Hct using the following equation (Dill and Costill, 1974):

$$\begin{array}{l} \%\Delta PV=[(Hb_{preex}/Hb_{postex})\;x\;[(100-Hct_{postex})/\\ \;(100-Hct_{preex})]-1]\;x\;100 \end{array}$$

Where ΔPV is percent change of PV, subscript a, is pre-iSPT; and subscript b, is post-iSPT.

A single-use rectal thermistor (Henleys, 400H, Henleys Medical, Welwyn Garden City) was used to measure rectal temperature (*T*_re_) from a depth of 10 cm past the anal sphincter and read by a connected data logger (Measurement, 4600, Henley-medical, Welwyn Garden City). Skin thermistors (Grant, EUS-U-VS5-0, Wessex-Power, Dorset) were attached to the right side of the body at the center of the pectoralis major, biceps brachi, rectus femoris, and gastrocnemius to measure skin temperature (*T*_*sk*_) with data recorded separately to a data logger (Eltek/Squirrel, Squirrel Series/model 451, Wessex Power, Dorset).

The following equation was used to calculate *T*_*sk*_ (Ramanathan, 1964).

$$\begin{array}{l} T_{sk}=0.3\left(T_{chest}+T_{arm}\right)+0.2\left(T_{thigh}+T_{calf}\right) \end{array}$$

Estimated muscle temperature (*T*_*mu*_) was also calculated using the following equation (Racinais et al., 2005a):

$$\begin{array}{l} T_{mu}=1.02\;x\;T_{sk}+0.89 \end{array}$$

Arterial blood oxygen saturation (S_a_O_2_) was measured via a finger pulse oximeter (Onyx® II 9550, Nonin-Medical, USA) fixed upon the participant's index finger. All body temperature measures (*T*_*re*_, *T*_*sk*_, and *T*_*mu*_), perceived exertion (RPE; Borg 6–20 scale; Borg, 1998) and thermal sensation (TS; 0–8 scale; Young et al., 1987) were collected in 15 min intervals.

### Statistical analysis

Normality of the observed data was assessed using quantile-quantile (Q—Q) plots and was deemed plausible in all instances with data presented as mean ± standard deviation (SD). Differences between condition, time, and condition x time for all physical and physiological measures were analyzed using linear mixed models (IBM-SPSS statistics for Windows, Version 21, Armonk, NY). This type of analysis was preferred as it (i) allows for missing data, (ii) can accurately model different covariate structures for repeated measures data, and (iii) can model between-subject variability (Vandenbogaerde and Hopkins, 2010; West et al., 2014). Where significance was obtained, Sidak *post-hoc* tests were used to locate significant pairs on all physical and physiological measures. A step down Hommel adjusted *post-hoc* pairwise comparisons was calculated for each physical and physiological measure if a significant main effect and/or interaction effect was present (Hommel, 1988). Two-tailed statistical significance was accepted at the *p* = < 0.05 level. The percentage changes between all physical performance measures are also reported and 95% CI presented where necessary. The most appropriate model was chosen using the smallest Hurvich and Tsai's criterion (AICC) in accordance with the principal of parsimony. Second, normality and homogeneity of variance of the residuals were checked using Q—Q plots and scatter plots, respectively, and deemed plausible in each instance. A Stepwise multiple linear regression analysis for each condition was performed in order to investigate which of the “physiological responses” (e.g., Body temperatures, Subjective and Physiological measures) were able to predict the environmentally-induced-decrements in physical performance (e.g., total distance, high-speed distance, sprint distance, and sprint distance covered).

## Results

### Physical performance

#### Overall and between halves

**Figure 2:**
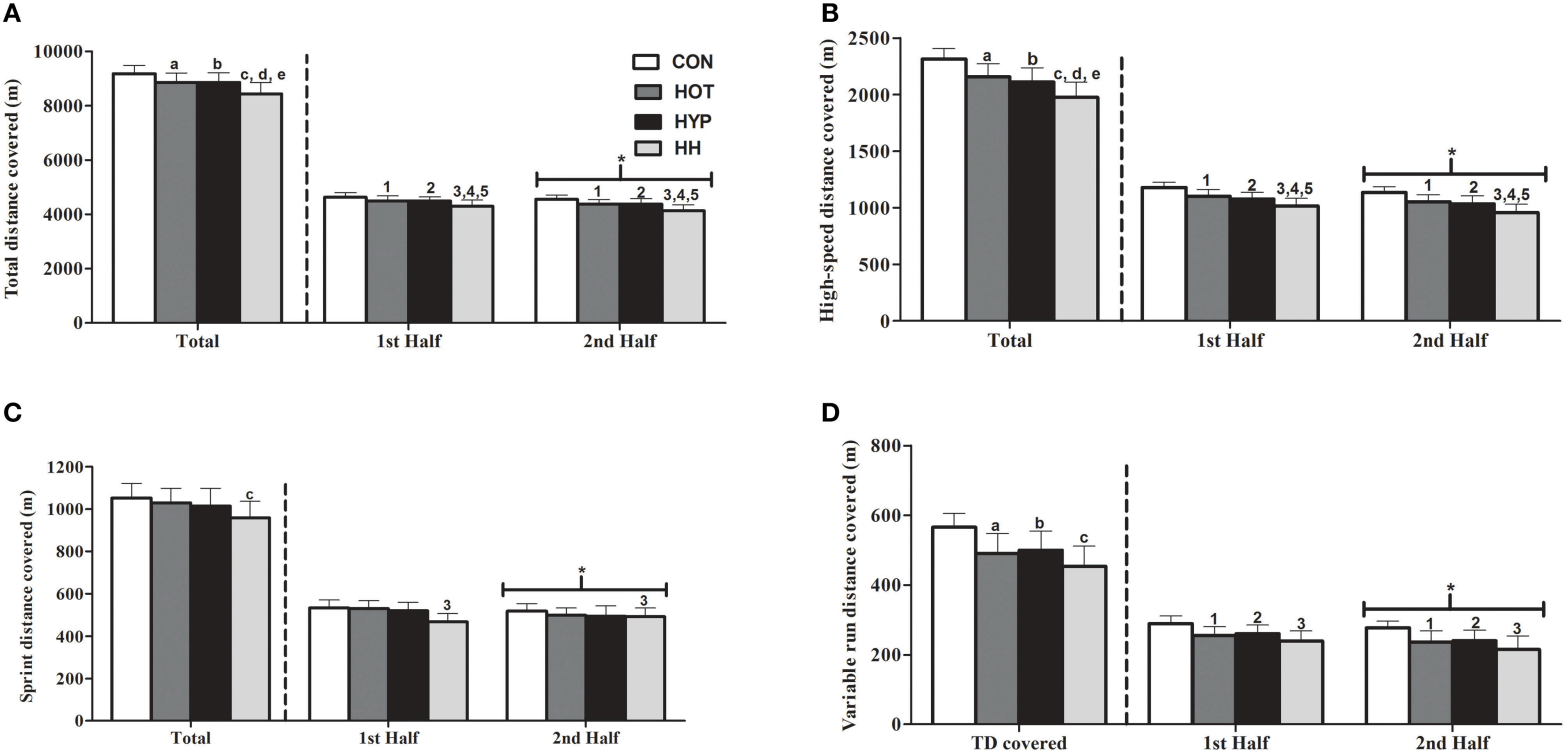
**The total distance covered (A), high-speed distance covered (B) variable run distance covered (C) and sprint distance covered (D) in total and in each half at CON, HOT, HYP, and HH**. Total, high-speed and variable run distance covered were significantly reduced (*p* < 0.05) in both halves of HOT and HYP compared with CON. These decrements for total and high-speed distance covered were exacerbated in HH compared with HOT and HYP. Sprint distance was significantly reduced (*p* < 0.05) in both halves of HH compared with CON. ^*^Significant difference from the first half;^a^Significant difference between CON and HOT (*p* < 0.05); ^b^Significant difference between CON and HYP (*p* < 0.05); ^*c*^Significant difference between CON and HH (*p* < 0.05); ^d^Significant difference between HOT and HH (*p* < 0.05); ^e^Significant difference between HYP and HH (*p* < 0.05);^1^Significant difference between halves in CON and HOT; ^2^Significant difference between halves in CON and HYP; ^3^Significant difference between halves in CON and HH ^4^Significant difference between halves in HOT and HH; ^5^Significant between halves in HYP and HH.

A significant main effect for condition (*F* = 16.5; *p* < 0.001), time (*F* = 202.8; *p* < 0.001), and an interaction effect for condition x time (*F* = 3.6; *p* = 0.03) was observed for total distance covered (Figure 2). Total distance covered was reduced by 4% in HOT (mean difference = 321 ± 131 m, *p* = 0.001, 95% CI: 65–256 m) and HYP (mean difference = 324 ± 136 m, *p* = 0.004, 95% CI: 44–282 m), and by 9% in HH (mean difference = 756 ± 142 m, *p* < 0.001, 95% CI: 196–560 m), compared to CON. A 5% reduction in total distance covered in HH compared to HOT (mean difference = 431 ± 132 m, *p* = 0.01, 95% CI: 41–395 m) and HYP (mean difference = 431 ± 132 m, *p* = 0.01, 95% CI: 41–395 m) was also evident. Between halves, the performance decrements were greater in HH (4%, mean difference = 164 ± 60 m, *p* < 0.001, 95% CI: 126–202 m), HYP (3%, mean difference = 101 ± 66 m, *p* < 0.001, 95% CI: 59–143 m), and HOT (2%, mean difference = 120 ± 45 m, *p* < 0.001, 95% CI: 91–148 m) compared with CON (1%, mean difference = 81 ± 66 m *p* = 0.001, 95% CI: 39–123 m). Furthermore, total distance covered was 3% (1st half) and 4% (2nd half) greater in CON compared to HOT (1st half: mean difference = 141 ± 53 m *p* = 0.007, 95% CI: 33–249 m, 2nd half: *p* = 0.001, 95% CI: 88–272 m) and HYP (1st half: mean difference = 152 ± 32 m *p* = 0.006, 95% CI: 34–271 m; 2nd half: *p* = 0.006, 95% CI: 41–305 m). Performance decrements in total distance covered were observed in HH compared to CON during the 1st (−8%, mean difference = 336 ± 32 m, *p* < 0.001, 95% CI: 144–529 m) and 2nd half (−10%, mean difference = 420 ± 63 m, *p* < 0.001, 95% CI: 242–597 m). A 4 and 6% reduction in total distance covered was also observed in the 1st and 2nd half in HH compared to HOT (1st half: mean difference = 184 ± 43 m, *p* = 0.04, 95% CI: 10–380 m; 2nd half: mean difference = 240 ± 32 m, *p* = 0.004, 95% CI: 68–412 m) and HYP (1st half: mean difference = 185 ± 33 m, *p* = 0.04, 95% CI: 10–381 m; 2nd half: mean difference = 243 ± 39 m, *p* = 0.04, 95% CI: 73–420 m), respectively (Figure 2).

A significant main effect for condition (*F* = 39.1; *p* < 0.001), time (*F* = 22.1; *p* < 0.001), and an interaction effect (*F* = 3.1; *p* = 0.04) was observed for high-speed distance covered (Figure 2). High-speed distance covered was reduced in HOT (−7%, mean difference = 160 ± 21 m, *p* = 0.001, 95% CI: 16–78 m), HYP (−9%, mean difference = 203 ± 32 m, *p* < 0.001, 95% CI: 62–81 m), and HH (−15%, mean difference = 340 ± 43 m, *p* < 0.001, 95% CI: 91–152 m) compared to CON. An 8% decrement in high-speed distance covered was observed in HH compared to HOT (mean difference = 180 ± 36 m, *p* < 0.001, 95% CI: 44–105 m) and HYP (mean difference = 182 ± 38 m, *p* < 0.001, 95% CI: 28–89 m). The performance decrements between halves was greater in HH (−6%, mean difference = 60 ± 30 m, *p* < 0.001, 95% CI: 126–202 m), HYP (−4%, mean difference = 46 ± 33 m, *p* = 0.003, 95% CI: 59–143 m), and HOT (−4%, mean difference = 48 ± 22 m, *p* < 0.001, 95% CI: 91–148 m) compared with CON (−3%, mean difference = 39 ± 16 m, *p* < 0.001, 95% CI: 39–123 m). Compared to CON, high-speed distance covered was reduced during the 1st half in HOT (−6%, mean difference = 76 ± 36 m, *p* = 0.002, 95% CI: 21–86 m), HYP (−8%, mean difference = 98 ± 64 m, *p* < 0.001, 95% CI: 23–110 m), and HH (−14%, mean difference = 160 ± 58 m, *p* < 0.001, 95% CI: 78–164 m). The high-speed distance covered was also reduced in the 2nd half in HOT (−7%, mean difference = 84 ± 58 m, *p* = 0.001, 95% CI: 7–94 m), HYP (−9%, mean difference = 105 ± 48 m, *p* = 0.001, 95% CI: 41–305 m), and HH (−16%, mean difference = 180 ± 68 m, *p* < 0.001, 95% CI: 78–165 m) compared to CON. Furthermore, a reduction in high-speed distance covered was evident at HH compared to HOT during the 1st (−8%, mean difference = 84 ± 47 m, *p* = 0.009, 95% CI: 35–121 m) and 2nd (−6%, mean difference = 96 ± 54 m, *p* = 0.009, 95% CI: 28–114 m) half. A decrement in high-speed distance covered was observed at HH compared to HYP during the 1st (−9%, mean difference = 61 ± 46 m, *p* = 0.007, 95% CI: 19–106 m) and 2nd (−7%, mean difference = 75 ± 54 m, *p* = 0.007, 95% CI: 12–98 m) half (Figure 2).

There was a significant main effect for condition (*F* = 4.8; *p* = 0.01), time (*F* = 92.6; *p* < 0.001), and an interaction effect (*F* = 3.7; *p* = 0.03) for sprint distance covered (Figure 2). The sprint distance covered was reduced in HH compared with CON (−8%, mean difference = 93 ± 36 m, *p* = 0.009, 95% CI: 9–83 m) and HOT (−7%, mean difference = 78 ± 46 m, *p* = 0.04, 95% CI: 7–69 m). The performance decrements between halves was greater in HH (−5%, mean difference = 24 ± 19 m, *p* = 0.001, 95% CI: 12–36 m), HYP (−5%, mean difference = 26 ± 24 m, *p* = 0.003, 95% CI: 11–41 m), and HOT (−6%, mean difference = 30 ± 17 m, *p* < 0.001, 95% CI: 20–41 m) compared with CON (−3%, mean difference = 15 ± 9 m, *p* < 0.001, 95% CI: 9–21 m). In CON, the sprint distance covered was greater in both halves (1st: −8%, mean difference = 38 ± 25 m, *p* = 0.04, 95% CI: 1.9, 81.5 m; 2nd: −10%, mean difference = 51 ± 35 m, *p* = 0.003, 95% CI: 14.2, 87.8 m) compared to HH (Figure 2).

There was a significant main effect for condition (*F* = 28.9; *p* < 0.001), time (*F* = 229.9; *p* < 0.001), and interaction effect (*F* = 5.8; *p* = 0.008) for variable run distance covered (Figure 2). The variable run distance covered was greater in CON compared with HOT (−13%, mean difference = 74 ± 24 m, *p* < 0.001, 95% CI: 22–53 m), HYP (−12%, mean difference = 65 ± 35 m, *p* < 0.001, 95% CI: 17–48 m), and HH (−15%, mean difference = 111 ± 37 m*, p* < 0.001, 95% CI: 34–78 m). The performance decrements between halves was greater in HH (−10%, mean difference = 24 ± 10 m, *p* < 0.001, 95% CI: 18–30 m), HYP (−8%, mean difference = 20 ± 10 m, *p* < 0.001, 95% CI: 14–27 m), and HOT (−7%, mean difference = 19 ± 7 m, *p* < 0.001, 95% CI: 14–23 m) compared with CON (−4%, mean difference = 12 ± 5 m, *p* < 0.001, 95% CI: 9–15 m). Variable run distance covered was greater in both halves of CON compared with HOT (1st: −10%, mean difference = 34 ± 30 m, *p* < 0.001, 95% CI: 20–48 m; 2nd: −15%, mean difference = 41 ± 38 m, *p* < 0.001, 95% CI: 22 59 m), HYP (1st: −9%, mean difference = 29 ± 23 m, *p* < 0.001, 95% CI: 14–43 m; 2nd: −13%, mean difference = 37 ± 25 m, *p* < 0.001, 95% CI: 18–55 m), and HH (1st: −17%, mean difference = 50 ± 35 m, *p* < 0.001, 95% CI: 28–72 m; 2nd: −22%, mean difference = 62 ± 31 m, *p* < 0.001, 95% CI: 38–89 m; Figure 2).

#### Between 15 min blocks

For high-speed distance covered, the performance decrements between the first and last 15 min blocks for CON (mean difference = 17 ± 6 m, *p* = 0.01, 95% CI: 3–21 m), HOT (mean difference = 31 ± 2 m, *p* = 0.001, 95% CI: 10–51 m), HYP (mean difference = 35 ± 7 m, *p* = 0.001, 95% CI: 11–55 m), and HH (mean difference = 49 ± 5 m, *p* = 0.001, 95% CI: 27–101 m) was −7, −8, −10, and −14%, respectively. The high-speed distance covered was reduced (*p* < 0.05) in all 15 min blocks in HOT [Range (%, m): −6 to −8%, 26–40 m], HYP [Range (%, m): −9 to −11%, 43–51 m], and HH [Range (%, m): −16 to −18%, 45–67 m] compared to CON (Table 3).

**Table 3:** **The HSD, SD, VRD covered and PSS in 15 min blocks during CON, HOT, HYP, and HH**.

	**0–15 min**	**15–30 min**	**30–45 min**	**45–60 min**	**60–75 min**	**75–90 min**
**HIGH-SPEED DISTANCE COVERED (m)**						
CON	400±15	393±17	384±17	388±14	378±18	373±21^*^
HOT	374±22^g^	366±20^g^	362±19^g^	359±22^g^	352±23^g^	343±24^*^^g^
HYP	367±20^h^	362±19^h^	351±24^h^	355±23^h^	347±24^h^	332±27^*^^h^
HH	355±24^i^	339±26^i^	324±30^i^	333±19^i^	319±20^i^	306±29^*^^i^
**SPRINT DISTANCE COVERED (m)**						
CON	182±13	177±12	174±12	174±13	175±11	170±12^*^
HOT	178±14	177±13	174±12	170±11	169±13	160±11^*^^g^
HYP	176±13	173±13	171±17	169±17	167±17	158±15^*^^h^
HH	171±16	164±17	157±14	162±12	157±12^i^	149±16^*^^i^
**VARIABLE RUN DISTANCE COVERED (m)**						
CON	100±8	95±7	94±9	95±7	92±7	90±7^^*^^
HOT	88±8	85±9	83±11	84±11	79±10	74±11^*^^g^
HYP	89±8	86±9	85±9	86±9	80±11	74±12^*^^h^
HH	84±10^i^	80±11^i^	76±10^i^	77±11^i^	71±11^i^	67±10^*^^i^
**PEAK SPRINT SPEED (km·h^−1^)**						
CON	21.5±1.2	21.1±1.7	20.2±1.6	19.8±1.3	21.1±1.7	21.5±1.8
HOT	22.1±1.5^g^^,^^j^^,^^k^	23.2±1.4^g^^,^^j^^,^^k^	23.2±1.5^g^^,^^j^^,^^k^	21.1±1.2^g^^,^^j^^,^^k^	22.1±1.3^g^^,^^j^^,^^k^	22.6±1.4^g^^,^^j^^,^^k^
HYP	21.1±1.3	22.1±1.2	22.6±1.8	20.7±1.5	21.2±1.5	21.5±1.9
HH	20.4±1.1	20.0±1.6	19.8±1.2	18.1±1.1	19.2±1.5	19.1±2.0

*The HSD, SD, and VRD covered are presented as an overall distance covered during each 15 min period. The PSS is presented as the fastest speed recorded in each 15 min period. CON, Normoxic-Temperate; HH, Hot-Hypoxic; HOT, Hot; HSD, High Speed Distance; Hyp, Hypoxic; PSS, Peak Sprint Speed SD, Sprint Distance; VRD, Variable Run Distance*;

* *Significant difference from the first 15 min*;

g *Significant difference in 15 min block between CON and HOT*;

h *Significant difference in 15 min block between CON and HYP*;

i *Significant difference in 15 min block between CON and HH*;

j *Significant difference in 15 min block between HOT and HYP*;

k *Significant difference in 15 min block between HOT and HH*.

The performance decrements for sprint distance covered between the first and 15 min block for CON (mean difference = 12 ± 14 m *p* = 0.007, 95% CI: 2–23 m), HOT (mean difference = 18 ± 12 m *p* = 0.005, 95% CI: 1–13 m), HYP (mean difference = 18 ± 12 m, *p* = 0.005, 95% CI: 2–15 m), and HH (mean difference = 22 ± 11 m, *p* < 0.001, 95% CI: −6 to −25 m) was −7, −11, −10, and 13%, respectively. A 6% decrease in sprint distance covered was observed in the final 15 min in CON compared with the identical time point in HOT (mean difference = 10 ± 13 m, *p* = 0.03, 95% CI: 1–20 m) and HYP (mean difference = 12 ± 21 m *p* = 0.03, 95% CI: 1–24 m). In CON compared with HH, the sprint distance covered was also increased by 9% (18 ± 12 m, *p* = 0.002, 95% CI: 6–30 m) and 12% (25 ± 11 m, *p* < 0.001, 95% CI: 9–33 m) in the final two 15 min blocks, respectively (Table 3).

The performance decrements between the first and last 15 min block in variable run distance covered for CON (mean difference = 10 ± 8 m, *p* = 0.04, 95% CI: 1–7 m), HOT (mean difference = 14 ± 9 m, *p* = 0.001, 95% CI: 4–18 m), HYP (mean difference = 15 ± 21 m, *p* = 0.04, 95% CI: 1–21 m), and HH (mean difference = 17 ± 21 m *p* = 0.04, 95% CI: 1–17 m) was 7, 8, 10, and 14%, respectively. The variable run distance covered was reduced (*p* < 0.05) in all 15 min blocks by ~18% [Range (%, m): 16–18%, 16–23 m] in HH compared to CON. An 8% decrease in variable run distance covered was seen in the final 15 min in CON compared with the identical time points in HOT (mean difference = 16 ± 12 m, *p* = 0.009, 95% CI: 2–17 m) and HYP (mean difference = 16 ± −13 m, *p* = 0.01, 95% CI: 2–17 m; Table 3).

The peak sprint speed reached in iSPT was 4% (3 ± 1 km·h^−1^), 4% (4 ± 1 km·h^−1^), and 7% (5 ± 1 km·h^−1^) faster in all 15 min blocks HOT than in CON (*p* = 0.03, 95% CI: 1–2 km·h^−1^), HYP (*p* = 0.03, 95% CI: 1–3 km·h^−1^), and HH (*p* = 0.03, 95% CI: 1–3 km·h^−1^), respectively. Furthermore, there was no significant difference (*p* > 0.05) in peak sprint speed between CON, HYP, and HH (Table 3).

### Body temperature

#### *T*_*re*_

**Figure 3:**
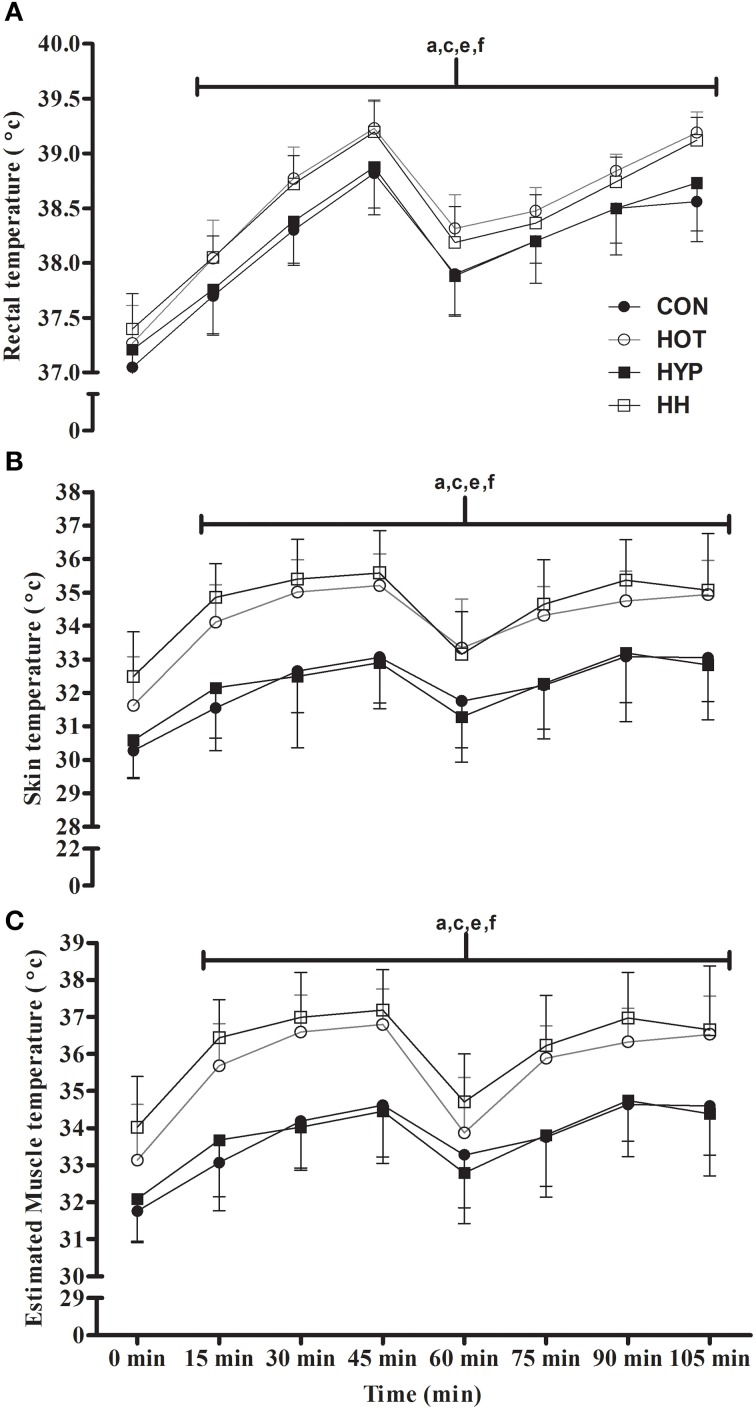
**The T_**re**_ (A), T_**sk**_ (B), and T_**mu**_ (C) during the first (0–45 min) and second (60–105 min) half in CON, HOT, HYP, and HH**. All body temperatures were significantly increased (*p* < 0.05) in HOT and HH compared with CON and HYP from 15–105 min. ^a^Significant difference between CON and HOT (*p* < 0.05); ^c^Significant difference between CON and HH (*p* < 0.05); ^e^Significant difference between HYP and HH (*p* < 0.05); ^f^Significant difference between HOT and HYP (*p* < 0.05).

There was a significant main effect for condition (*F* = 4576.7; *p* < 0.001), time (*F* = 12.9; *p* < 0.001), and an interaction effect (*F* = 2.2; *p* = 0.007) for *T*_*re*_. The mean *T*_*re*_ in HOT (38.7 ± 0.2°C) was elevated by 2% compared with both CON (38.3 ± 0.3°C, *p* < 0.001, 95% CI: 0.2–0.5°C) and HYP (38.3 ± 0.4°C, *p* = 0.001, 95% CI: 0.1–0.4°C). Furthermore, the mean T_re_ in HH (38.6 ± 0.2°C) was also increased (2%) when compared with both CON (*p* = 0.001, 95% CI: 0.1–0.6°C) and HYP (*p* = 0.009, 95% CI: 0.1–0.4°C). There was no significant difference (*p* = 1.000, 95% CI: −0.2 to 0.2°C) in mean *T*_*re*_ between HOT and HH. At all-time points including and after 15 min, *T*_*re*_ was significantly increased (*p* < 0.001) in HOT and HH compared with CON and HYP (Figure 3).

#### *T*_*sk*_

There was a significant main effect for condition (*F* = 2163.7; *p* < 0.001), time (*F* = 40.9; *p* < 0.001), and main effect for condition x time (*F* = 28.9; *p* < 0.001) for *T*_*sk*_. The mean *T*_*sk*_ in HOT (34.1 ± 1.0°C) was elevated by 5% compared with both CON (32.5 ± 1.3 °C, *p* < 0.001, 95% CI: 1–3°C) and HYP (32.4 ± 1.5 °C, *p* < 0.001, 95% CI: 1–2°C). Furthermore, the mean *T*_*sk*_ in HH (34.5 ± 1.2°C) was also increased (5%) when compared with both CON (*p* < 0.001, 95% CI: 1–3°C) and HYP (*p* < 0.001, 95% CI: 1–3°C). There was no significant difference (*p* = 1.000, 95% CI: −1.8 to 0.6°C) in mean *T*_*sk*_ between HOT and HH. At all-time points including and after 15 min, *T*_*sk*_ was significantly increased (*p* < 0.001) in HOT and HH compared with CON and HYP (Figure 3).

#### Estimated *T*_*mu*_

There was a significant main effect for condition (*F* = 2163.7; *p* < 0.001), time (*F* = 40.9; *p* < 0.001) and an interaction effect (*F* = 28.9; *p* < 0.001) for *T*_*mu*_. The mean estimated *T*_*mu*_ in HOT (35.7 ± 1.0°C) was elevated by 5% compared with both CON (34.1 ± 1.3 °C, *p* < 0.001, 95% CI: 1–2 °C) and HYP (33.9 ± 1.5°C, *p* < 0.001, 95% CI: 1–2°C). Furthermore, the mean estimated *T*_*mu*_ in HH (36.1 ± 1.2°C) was also increased (5%) when compared with both CON (*p* < 0.001, 95% CI: 1–3°C) and HYP (*p* < 0.001, 95% CI: 1–3°C). There was no significant difference (*p* = 1.000, 95% CI: −1.7 to 0.6°C) in mean estimated *T*_*mu*_ between HOT and HH. At all-time points including and after 15 min, estimated *T*_*mu*_ was significantly increased (*p* < 0.001) in HOT and HH compared with CON and HYP (Figure 3).

### Subjective measures

**Figure 4:**
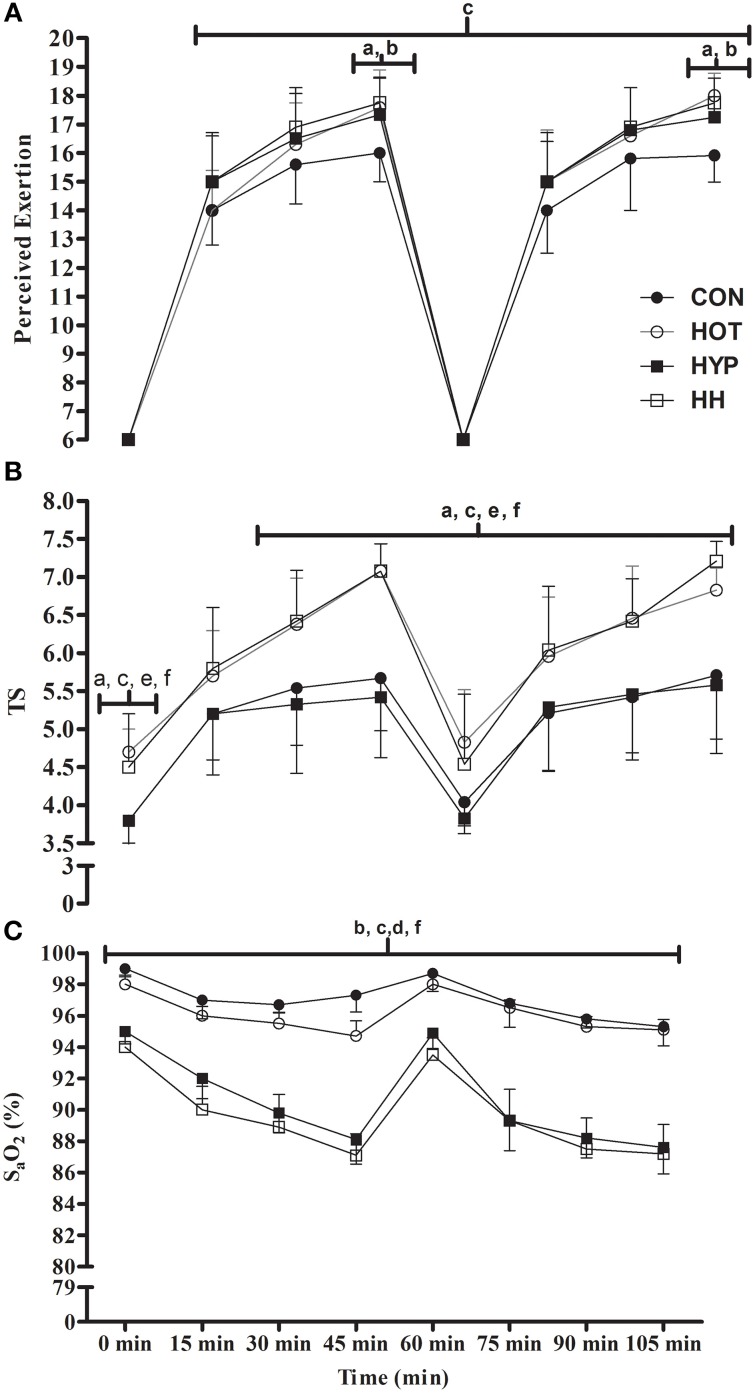
**The Perceived Exertion (A) and TS (B) and S_**a**_O_**2**_ (C) during the first (0–45 min) and second (60–105 min) half in CON, HOT, HYP, and HH**. Perceived exertion was significantly increased from 30–105 min in HOT, HYP, and HH compared with CON. A significant increase in TS was evident at 0 min and 30–105 min in HOT and HH compared with CON and HYP. Furthermore, S_a_O_2_ was significantly reduced in from 15–105 min in HYP and HH compared with CON and HOT. ^a^Significant difference between CON and HOT (*p* < 0.05); ^b^Significant difference between CON and HYP (*p* < 0.05); ^*c*^Significant difference between CON and HH (*p* < 0.05); ^d^Significant difference between HOT and HH (*p* < 0.05); ^e^Significant difference between HYP and HH (*p* < 0.05);^f^Significant difference between HOT and HYP (*p* < 0.05).

There was a significant main effect for condition (*F* = 20.8; *p* < 0.001), time (*F* = 1140.3; *p* < 0.001), and an interaction effect (*F* = 1.8; *p* = 0.02) for RPE (Figure 4). Perceived Exertion was 7% lower during CON (15 ± 2) compared with HOT (16 ± 2, *p* < 0.001, 95% CI: 0–1), HYP (16 ± 2, *p* < 0.001, 95% CI: 0–1), and HH (17 ± 1, *p* < 0.001, 95% CI: 1–2). Perceived Exertion was greater (*p* < 0.05) in HH compared to CON from all-time points after 15 min, and increased at 45 and 105 min in HOT (*45 min*: *p* < 0.001, 95% CI: 1–3; *105 min*: *p* < 0.001. 95% CI: 1–3) and HYP (*45 min*: *p* = 0.001. 95% CI: 1–3; *105 min*: *p* = 0.006, 95% CI: 1–3) compared to CON (Figure 4).

Figure 4 reveals a significant main effect for condition (*F* = 96.5; *p* < 0.001), time (*F* = 106.2; *p* < 0.001) and an interaction effect (*F* = 1.8; *p* = 0.01) for TS. The TS was 18% lower during CON (5 ± 1) and HYP (5 ± 1) compared with HOT (6 ± 1) (CON: *p* < 0.001, 95% CI: 1–2; HYP: *p* < 0.001, 95% CI: 1–2) and HH (6 ± 1) (CON: *p* < 0.001, 95% CI: 1–2; HYP: *p* < 0.001, 95% CI: 1–2). A significant increase (*p* < 0.05) in TS during HOT and HH at 0 and 30–105 min compared with CON and HYP (Figure 4).

### Arterial blood oxygen saturation

There was a significant main effect for condition (*F* = 453.8; *p* < 0.001), time (*F* = 133.4; *p* < 0.001), and an interaction effect (*F* = 12.2; *p* < 0.001) for S_a_O_2_. Mean S_a_O_2_ was 97.4, 96.9, 90.5, and 89.4% in CON, HOT, HYP, and HH, respectively. During HYP and HH a 7% decrease in S_a_O_2_ was evident compared with CON (HYP: *p* < 0.001, 95% CI: 6–8%; HH: *p* < 0.001, 95% CI: 7–9) and HOT (HYP: *p* < 0.001, 95% CI: 5–6%; HH: *p* < 0.001, 95% CI: 6–7%). A significant reduction (*p* < 0.05) in S_a_O_2_ was also seen during HYP and HH compared with CON and HOT at all-time points (Figure 4).

### Heart rate response

There was a significant main effect for condition (*F* = 5.8; *p* = 0.004), but there was no significant main effect for time (*F* = 1.3; *p* = 0.28) and no interaction effect (*F* = 0.1; *p* = 0.99) for HR. Mean HR during CON, HOT, HYP, and HH was 161 ± 10, 163 ± 3, 165 ± 7, and 168 ± 8 b·min^−1^, respectively. In HH, a significant increase (7 ± 11 b·min^−1^, *p* < 0.001, 95% CI: 1–13 b·min^−1^) by 4% was seen compared with CON. Furthermore, The HR was also increased (4 ± 9 b·min^−1^*, p* = 0.002, 95% CI: 2–13 b·min^−1^) by 3% in HYP compared with CON. No significant change (2 ± 9 b·min^−1^, *p* = 0.30, 95% CI: −2 to 8 b·min^−1^) in HR was seen between CON and HOT.

### Body mass changes

There was a significant main effect for condition (*F* = 10.8; *p* < 0.001), time (*F* = 162.5; *p* < 0.001), and an interaction effect (*F* = 2.9; *p* = 0.04) for body mass. Body mass was significantly reduced post-iSPT by 2% (2 ± 1 kg) in both HOT (HOT vs. CON: 75 ± 12 kg, *p* < 0.001, 95% CI: 1–2 kg; HOT vs. HYP: *p* < 0.001, 95% CI: 1–2 kg) and HH (HH vs. CON: 75.6 ± 11.2 kg, *p* = 0.005, 95% CI: 0–2 kg; HH vs. HYP: *p* = 0.005, 95% CI: 0–2 kg) compared to CON (77 ± 11 kg) and HYP (77 ± 11 kg).

### Blood lactate and plasma volume changes

#### Bla concentration

There was a significant main effect for condition (*F* = 18.4; *p* < 0.001) and time (*F* = 90.1; *p* < 0.001), for Bla. However, no interaction effect (*F* = 0.7; *p* = 0.77) was evident between halves and individual time points for Bla. Between conditions, the Bla concentration at HH was only significantly increased (1.5 mmol^−1^, *p* < 0.001, 95% CI: 1–2 mmol^−1^) compared with CON. No significant difference (*p* < 0.05) in Bla concentration was evident between CON, HOT, and HYP (Table 4).

**Table 4:** **The Bla concentration and plasma volume changes at each individual time point, half and total during CON, HOT, HYP, and HH. The Bla concentration is presented in mmol^**−1**^**.

	**0 min**	**12 min**	**27 min**	**45 min**	**1st half**	**57 min**	**72 min**	**90 min**	**2nd half**	**Total**
**BLA CONCENTRATION (mmol^−1^)**										
CON	0.9 ± 0.3	4.8 ± 1.0	4.6 ± 1.0	4.7 ± 1.1	4.6 ± 1.0	4.3 ± 1.4	4.0 ± 1.5	3.3 ± 1.3	3.9 ± 1.3	4.3 ± 1.3
HOT	0.9 ± 0.3	5.1 ± 1.8	5.4 ± 1.4	4.1 ± 1.7	5.1 ± 1.5	3.9 ± 1.6	4.7 ± 2.0	3.3 ± 1.5	4.0 ± 1.3	4.4 ± 1.9
HYP	0.8 ± 0.2	5.3 ± 1.0	5.3 ± 1.1	4.4 ± 1.7	5.1 ± 1.1	4.0 ± 1.9	4.3 ± 1.5	3.3 ± 0.9	3.8 ± 1.2	4.5 ± 1.6
HH	0.9 ± 0.3	6.7 ± 1.1	6.0 ± 1.4	5.5 ± 1.5	6.0 ± 1.0	5.7 ± 1.2	5.7 ± 1.3	5.0 ± 1.3	5.6 ± 1.0	5.8 ± 1.8^c^
**PLASMA VOLUME CHANGE (%)**										
CON	0 ± 0	–	–	–	–	–	–	–	–	−2.3±1.2
HOT	0 ± 0	–	–	–	–	–	–	–	–	−3.1±1.5
HYP	0 ± 0	–	–	–	–	–	–	–	–	−3.1±1.7
HH	0 ± 0	–	–	–	–	–	–	–	–	−7.2±2.2^c^

*Plasma volume change is presented as a percentage (%) change between pre- and post-iSPT. Bla, Blood lactate; CON, Normoxic-Temperate; HH, Hot-Hypoxic; HOT, Hot; Hyp, Hypoxic*;

c *Significant difference between CON and HH (p < 0.05)*.

#### Plasma volume change

There was also a significant main effect for condition (*F* = 20.2; *p* < 0.001), time (*F* = 88.6; *p* < 0.001), and interaction effect (*F* = 0.9; *p* = 0.04) for plasma volume change. Between pre- and post-iSPT, there was a significant reduction in plasma volume change in CON (*p* = 0.001, 95% CI = −1 to −3%), HOT (*p* < 0.001, 95% CI = −1 to −5%), HYP (*p* < 0.001, 95% CI = −1 to −4%), and HH (*p* < 0.001, 95% CI = −3 to −11%), between pre- and post-iSPT. In HH, a significantly greater reduction (*p* < 0.001, 95% CI: −3 to −7%) in plasma volume change was evident compared with CON (Table 4).

### Regression analysis

A stepwise regression analysis identified that absolute TS at the end of HOT was a predictor of the total distance (*r* = 0.82, *p* = 0.05) and high-speed distance covered (*r* = 0.82, *p* = 0.05) during the HOT condition. The absolute rise from the start to end of HOT for *T*_*mu*_ (*r* = 0.84, *p* = 0.02) and *T*_*sk*_ (*r* = 0.82, *p* = 0.02) was also a predictor for the total distance and high-speed distance covered at HOT. The absolute TS during HOT was also a predictor of the percentage reduction (5%) for the total distance covered (*r* = 0.82, *p* = 0.02) from CON to HOT. No other physiological measures were found to be significant predictors of the physical performance decrements seen in HYP and HH.

## Discussion

The present study examined the changes in simulated soccer performance in HOT, HYP, and HH conditions compared with CON, by utilizing the recently validated iSPT (Aldous et al., 2014). The main finding revealed a marked decline in total distance, high-speed distance, and variable run distance covered during HOT, HYP, and HH conditions when compared to CON (Figure 2), supporting the first experimental hypothesis. A secondary finding was that peak sprint speed, was increased in HOT compared with CON, HYP, and HH and that sprint distance covered was unchanged in HOT and HYP, supporting the second experimental hypothesis (Figure 2 and Table 3). Furthermore, a greater decline in physical performance was seen in HH even though physiological changes in body mass and temperatures (Figure 3), HR, subjective measures (Figure 4) and S_a_O_2_ (Figure 4) were not exacerbated compared to HOT and HYP. This change in physical performance was likely due to alterations in Bla concentration and plasma volume which were only present in HH, supporting the third experimental hypothesis.

The data from this study reveals a 4% reduction in total distance and high-speed distance covered in both HOT and HYP compared with CON, which agrees with previous match-play studies in the heat (43°C; Mohr et al., 2012) and at low altitudes (1600 m; Garvican et al., 2014). The performance decrements for total distance, high-speed distance and variable run distance covered between halves (Figure 2) were greater in HOT (8–11%), HYP (10%), and HH (13–14%) compared to CON (7%). In contrast to our results, Mohr et al. (2012) reported the performance decrements between halves was greater in temperate (21°C) compared to hot (43°C) conditions during match-play. This increased performance decrement is indicative of an adaptive match-play-specific pacing strategy which is postulated to preserve technical skill execution (Mohr et al., 2012; Nassis, 2013; Nassis et al., 2015). The environmental stress may likely reduce the “willingness” of an athlete to perform physical exercise during match-play (Mohr et al., 2012; Aughey et al., 2013). The iSPT (Aldous et al., 2014) prevents adoption of these pacing strategies (i.e., match factors; Gregson et al., 2010) with the same exercise performed in each half due to the individualized and externally-controlled speed thresholds. Therefore, players cannot preserve their sprinting characteristics during iSPT by minimizing their high-speed activity as observed during soccer match play (Nassis et al., 2015).

A participants “willingness” to perform high-speed exercise at a self-paced speed was measured during iSPT, via the variable run component, which is designed to quantify high-speed running without an external cue (Aldous et al., 2014). However, when these external cues are removed in the variable run, participants choose a lower running speed in HOT, HYP, and HH compared to CON, which might be indicative of the environment-mediated performance decrements observed in soccer match play (Mohr et al., 2012; Garvican et al., 2014). Furthermore, significant reductions in variable run distance covered in HOT, HYP, and HH both between halves (Figure 2) and in the final 15 min compared with CON (Table 3) were observed. Conversely to soccer match-play at 43°C (Mohr et al., 2012) the performance decrements (high-speed distance, sprint distance, and variable run distance) between the first and last 15 min block was increased in HOT, HYP, and HH when compared with CON (Table 3); likely due to iSPT controlling pacing and match factors (Aldous et al., 2014). This decline in variable run distance supports the notion that the individualized externally-controlled movement patterns employed by iSPT prevented participants adopting an altered pacing strategy. However, previous soccer match-play data has identified that soccer players can preserve key physical performance measures (e.g., sprint distance covered) in hot and hypoxic environments (Nassis, 2013; Nassis et al., 2015), yet decrements in high-speed and sprint distance covered still occur in the final 15 min of match-play (76–90 min) when compared to the first 15 min (0–15 min; (Mohr et al., 2010, 2012)). These performance impairments may influence the match outcome as a number of studies have revealed more goals are scored/conceded in the final 15 min (76–90 min) of match-play (Abt et al., 2002; Armatas et al., 2007). This phenomenon in goals scored/conceded is likely due to an inability to maintain repeated sprint exercise or discrete episodes of non-fatigued maximal physical performance [central to match outcome (Faude et al., 2012)], within the final 15 min of match-play (Gregson et al., 2010; Faude et al., 2012) as supported by the presented data (Figure 2).

A further finding from the present study was that sprint distance covered was unchanged in HYP and HOT, however, peak sprint speed was also improved in HOT compared with CON, showing synergy with previous match-play data (Mohr et al., 2012; Nassis, 2013; Nassis et al., 2015). In HOT, the increase in peak sprint speed could be explained by an increase in estimated *T*_*mu*_ which has been shown to improve muscle contractile properties (Racinais et al., 2004), leading to a higher power production and in turn a better sprint performance (Racinais et al., 2005b). However, improvements in sprint performance during soccer match-play in hot environments has been only shown to occur when T_re_ is below 39°C (Mohr et al., 2012). Therefore, this could explain the significant reduction in sprint distance covered in the last 15 min in HOT (Table 3). Furthermore, Nassis (2013) identified that elite soccer players in the 2010 FIFA World Cup were able to preserve their peak sprint speed across match-play at low altitudes due to the altered composition to the atmosphere (i.e., air being thinner) which improves the aerodynamics and flight time of an athlete through the air (Levine et al., 2008). However, a hypobaric chamber was not available during this study, so a hypoxicator mask was used to simulate a low altitude environment despite the larger energy cost required when these types of masks are worn (Coppel et al., 2015). The mask was worn in all four experimental conditions to control for this potential confounding factor. Previous research has identified single and repeated sprint performance is maintained at altitude due to a greater anaerobic energy release (Calbet et al., 2003; Morales-Alamo et al., 2012). This is due to several metabolic pathways being stimulated to supplement energy production when aerobic metabolism is not capable of matching aerobic ATP production to consumption, especially the splitting of phosphocreatine (PCr) and glycolysis (Calbet et al., 2003). However, this is likely to manifest itself as a greater and earlier onset of fatigue toward the end of prolonged high-speed exercise as an increase in muscle lactate accumulation would account for a reduction in aerobic ATP production (Balsom et al., 1994; Billaut and Smith, 2010). Therefore, this could explain the exacerbated decline in sprint distance covered during the final 15 min in HYP (Table 3).

Despite similar decrements in physical performance in both HOT and HYP compared to CON, the physiological underpinning of such responses differ. Elevated *T*_*re*_, *T*_*sk*_, and estimated *T*_*mu*_ (Figure 2) in HOT and HH were seen from 15 min onwards compared with CON and HYP, showing parity with previous soccer match-play research (Mohr et al., 2010, 2012; Özgünen et al., 2010). In HOT, the absolute rise in *T*_*sk*_ and estimated *T*_*mu*_ predicted total and high-speed distance covered, with end TS predicting the decrement in total distance. As both *T*_*sk*_ and TS have a strong relationship (Sawka et al., 2012), thermal comfort is likely central to the physical performance decrements seen in HOT. Interventions should target these specific factors (*T*_*sk*_, estimated *T*_*mu*_ and TS) in an attempt to maintain “temperate-like” match play soccer performance.

An increase in HR at HYP when compared with CON, shows synergy with previous soccer match-play data at 1600 m above sea level (Garvican et al., 2014). The rise in HR seen in HYP can be attributed to a hemodynamic response arising from a reduction in S_a_O_2_ which drives a compensatory increase in cardiac output (Mazzeo, 2008; Stembridge et al., 2015a,b). However, during high-speed exercise bouts at altitude a decrease in stroke volume can decrease O_2_ delivery to the active muscles as it cannot match the muscle demand, manifesting as a decline to physical performance in HYP (Mazzeo, 2008). A reduction in S_a_O_2_ by ~8% compared to CON was also apparent by the end of iSPT in both HYP and HH which indicates the onset of exercise induced arterial hypoxemia had occurred causing a plethora of detrimental physiological responses (Billaut and Aughey, 2013), driving the exacerbated performance decrements seen in HYP and HH (Figure 2). Indeed, reduced phosphocreatine re-synthesis at altitude is due to sub-optimal re-oxygenation of the active skeletal muscle elongating the recovery time between high-speed exercise bouts (Garvican et al., 2014). Changes in high-speed running are important for maintaining match-play physical performance, due to its association with game defining moments (Gregson et al., 2010), possibly impacting upon the match result (Taylor and Rollo, 2014). Furthermore, the employed design cannot distinguish precisely between whether the changes in S_a_O_2_ were apparent due to exercise and/or environmentally-induced-arterial-hypoxemia, highlighting that future work should look to explore these complex phenomena within an appropriate design. Data by Billaut and Smith (2010) indicates that intermittent running based exercise can induce exercise-induced-arterial-hypoxemia in University level soccer players. Therefore, although the employed design cannot distinguish precisely between exercise and environmentally-mediated-arterial-hypoxemia future work should look to explore these complex phenomena within an appropriate design.

In HH, the largest performance decrement both between halves (Figure 2) and 15 min blocks was evident (Table 3). However, all changes in TS (Figure 4), body mass and temperature (Figure 3) were similar compared with HOT. Furthermore, all changes to both S_a_O_2_ (Figure 4) and HR were comparable with HYP. This is despite a greater decline in total distance and high-speed distance covered, as well as an additional reduction in sprint distance covered in HH which were not present in HOT and HYP (Figure 2). This exacerbated reduction to physical performance in HH may have been due to a significant increase in Bla concentration which may indicate a greater anaerobic energy release compared with CON, HOT, and HYP (Amann et al., 2006). Furthermore, a 5% reduction in plasma volume (Table 3) which coincided with a 2% change in body mass post-iSPT in HH may have meant that the participants finished iSPT in a hypo-hydrated state, (Cheuvront et al., 2003) causing an increase to the rate of heat storage and sweat output which in turn can impair prolonged high-speed activities in hot environments (Cheuvront and Kenefick, 2014). Additionally, HR was also increased during HH, showing parity with previous research in a hot and low altitude environment (30°C; 1900 m; Buono et al., 2012). This augmented HR response in HH likely stemmed from an impaired stroke volume and/or cardiac output, previously seen during prolonged exercise bouts in heat (González-Alonso et al., 2008) and hypoxia (Mazzeo, 2008). Thus, the exacerbated decline in performance was likely caused by a combination of both hot and hypoxic-mediated fatigue mechanisms. It is already acknowledged that both heat and hypoxia induce performance decrements via these mechanisms during soccer match-play likely influencing match outcome (Taylor and Rollo, 2014). Therefore, the number of game defining moments may be further decreased within HH.

The use of recreationally active male volunteers, rather than elite soccer players, is a limitation of this study; so any generalization of the results to such populations should be considered cautiously. However, our sample included participants with a $\dot{\text{V}}{\text{O}}_{2\text{max}}>$55 mL^.^kg^−1.^min^−1^, demonstrating some parity with elite soccer (Tonnessen et al., 2013). The assessment of technical skills and multi-directional movements were unable to be quantified by iSPT (Aldous et al., 2014). Therefore, to assess these within a similarly valid soccer-specific simulation, match factors and protective adaptive pacing strategies must be controlled, in order to robustly assess whether technical skills would remain unchanged in line with previous match-play data (Mohr et al., 2012; Nassis, 2013; Nassis et al., 2015).

The data from this study can be utilized to ascertain the efficacy of any ergogenic intervention to offset the environmentally-induced-decrements. For example, pre- and/or half-time-cooling has been reported to have an ergogenic effect upon both aerobic (Duffield et al., 2010) and repeated-sprint performance in the heat (Castle et al., 2006). Dietary nitrate has also been shown to improve muscle oxygenation during sub-maximal and maximal exercise in acute severe hypoxia (Masschelein et al., 2012; Wylie et al., 2013; Thompson et al., 2015). Furthermore, key physical performance measures (e.g., high-speed distance and sprint distance covered) associated with the match outcome in soccer (Gregson et al., 2010; Faude et al., 2012) are impaired in hot, hypoxic and hot-hypoxic environments, potentially decreasing the number of game defining events during match-play (Taylor and Rollo, 2014). Therefore, the efficacy of these interventions may be important for practitioners and governing bodies to attenuate these decrements present for key physical performance measures during soccer match-play in hot and hypoxic environments.

In conclusion, the present study shows that during simulated soccer performance, total distance, high-speed distance and variable run distance covered are significantly impaired within hot (30°C), hypoxic (1000 m above sea level), and hot-hypoxic (30°C; 1000 m above sea level) conditions when compared to a normoxic-temperate environment. Furthermore, peak sprint speed, was increased in HOT compared with CON, HYP, and HH. However, sprint distance covered was unchanged in HOT and HYP and only decreased in HH compared with CON. It is also revealed that the reduction in soccer physical performance is exacerbated in HH, compared to HOT and HYP alone. The heat-induced-decrements in HOT stem from increasing body temperatures, TS and the 2% reduction in body mass. The hypoxic-induced-decrements in HYP were most likely initiated by a decrease in SaO2 and increase in HR. Similar changes in TS, body mass and temperatures were seen in HOT compared with HH, whilst similar changes in HR and SaO2 were evident in HH compared to HYP. Furthermore, both Bla and plasma volume change alterations were only seen in HH compared with CON, highlighting that both these measures may play a role in the exacerbated decrements seen in HH. However, a deductive design to assess whether simulated soccer performance would still decrease in HH if plasma volume was maintained is needed to understand the mechanistic cause of these findings. The aforementioned physiological changes seen in the present study may influence the decrements to physical performance seen in HOT, HYP, and HH. Therefore, a detrimental effect on the match outcome may be seen in soccer match-play in these environments, which would be important to practitioners within soccer.

## Author contributions

Conceived and designed the experiments JA, BC, IA, BD, GA, and LT. Performed the experiments JA, BC, IA, BD, GA, and LT. Analyzed the data JA, BC, IA, BD, GA, and LT. Contributed reagents/materials/analysis tools JA, BC, IA, BD, GA, and LT. Wrote the paper JA, BC, IA, BD, GA, and LT.

## Funding

This research was funded by the João Havelange Research Scholarship on behalf of the Fédération Internationale de Football Association (FIFA). No commercial or financial incentives were provided that can have caused any potential conflict of interest. The authors would like to thank the participants for their involvement in this study. There was no conflict of interest for any author in this study.

### Conflict of interest statement

The authors declare that the research was conducted in the absence of any commercial or financial relationships that could be construed as a potential conflict of interest.
